# Macromolecular and Supramolecular Organization of Ionomers

**DOI:** 10.3390/polym17233188

**Published:** 2025-11-29

**Authors:** Ilsiya M. Davletbaeva, Oleg O. Sazonov

**Affiliations:** Technology of Synthetic Rubber Department, Kazan National Research Technological University, 68 Karl Marx str., Kazan 420015, Russia; sazonov.oleg2010@gmail.com

**Keywords:** ionomers, synthesis, macromolecular structure, supramolecular organization, ionic clusters

## Abstract

Ionomers are promising materials because ionic interactions and their reversible clustering provide sensitivity to stimuli and facilitate energy dissipation, polymer miscibility, and ion transport. The existence of a wide variety of interacting ionic groups and their associated macromolecular structures provides the basis for considering the supramolecular organization of ionic polymeric materials as a factor determining the emergence of specific properties. The main structural elements of ionomers are ionic clusters, and the properties of ionomers are determined by their sizes and size distribution. Ionomers are attractive for use in composites, actuators, coatings, dyed textiles, adhesives, shape-memory and self-healing materials, water purification membranes, and ion-exchange membranes for fuel cells and batteries. This paper presents a review of the macromolecular structure and supramolecular organization of ionomers and their properties, depending on the basis of their ionic functionalization. The ionic functions of ionomers are determined primarily by the type of ion (cations or anions) that serves as the basis for their functionalization. Ionomers containing both anionic and cationic pendant ions are considered, with attention given to the influence of the nature of the counterions used on the properties of ionomers.

## 1. The Basic Characteristics of Ionomers

Ionic polymeric materials differ fundamentally from polymers, whose macrochains are constructed solely by covalent bonding [1]. Such materials include ionomers and polyelectrolytes, which differ fundamentally in the content of ionic groups in their structures. Ionic polymeric materials also include ionic hydrogels, which consist of ionomers or polyelectrolytes but, unlike them, are physically or chemically cross-linked networks capable of not only swelling in water but also retaining large quantities of water. Polymers coordinately bound by transition metal ions can also exhibit characteristics of ionic polymeric materials.

A distinctive feature of the chemical structure of ***ionomers*** is the content of ionic groups in the side branches, ranging from 1 to 15 mol.%. One of the first commercially produced ionomers was a poly(ethylene-co-methacrylic acid)-based thermoplastic developed by DuPont™ under the name Surlyn^®^ and studied in [2,3,4,5]. Due to the high mobility of ions within ion clusters, Surlyn^®^ responds reversibly to temperature, contracting during heating and, conversely, expanding during cooling [6,7]. These materials are widely known for their exceptional transparency, strength, durability and self-healing ability. Another commercially available ionomer, Nafion^®^, was developed based on polytetrafluoroethylene containing terminal sulfonate chains and is used primarily as a proton conductor for proton exchange membranes [8,9,10]. In addition to Surlyn^®^ and Nafion^®^, a commercially used ionomer is Hypalon, a thermoplastic elastomer produced from chlorosulfonated polyethylene [11,12]. Hypalon ionomers are characterized by high chemical resistance and resistance to UV radiation, so these polymers are widely used in the construction industry for the production of roofing materials and consumer goods, in particular inflatable rafts.

This review focuses on the features of the chemical and supramolecular structure of ionomers, as well as the resulting mechanical and physicochemical properties and areas of practical use of such polymeric materials. The presence of labile ionic interactions in the structure of ionomers allows them to be classified as dynamic materials. The binding energies of ionic compounds are lower than the covalent bond energies, which, under certain conditions, enable their reversible destruction and restoration and create the preconditions for the formation of new and unusual supramolecular structures.

The existence of a wide variety of interacting ionic groups and associated macromolecular structures is the basis for considering the supramolecular organization of ionomers as a factor determining the emergence of specific properties [12,13]. Supramolecular structures in polymeric materials arise as a result of hydrogen bonding of different structural macromolecular segments or π-π interactions, coordination interactions with metal ions, ionic bonding, and host–guest interactions. Combinations of several temporary bonds are possible. For controlling the supramolecular structure of polymers, fundamental importance is attached to their molecular conformations [14,15,16], chain microstructure, and the ability to place functional groups along the linear backbone at precisely periodic intervals [17,18,19,20]. Dynamic covalent bonds in cross-linked polymer environments are realized in covalent adaptable networks, in which the exchange of such bonds occurs under mild conditions or is controlled by periodic stimuli such as heating [21,22]. Activation of bond exchange in the polymer matrix leads to stress relaxation and topological changes, and the process itself is usually divided into dissociative and associative mechanisms [23]. Due to the ability to dynamically activate bond exchange, covalent adaptable networks exhibit exceptional strength, self-healing ability, and shape memory [24,25,26,27,28,29,30,31].

**Figure 1:**
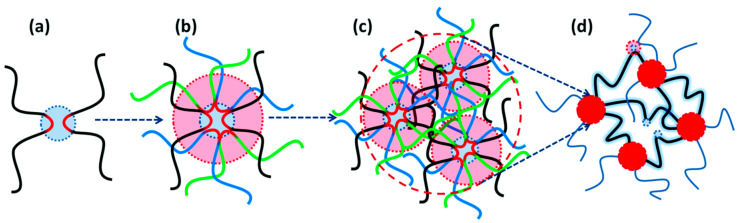
Schematic representation of the phase-separation process in supramolecular polymeric materials: (**a**) doublet connection; (**b**) aggregation of doublets into larger multiplets is limited by the necessary extension of surrounding chain segments; (**c**) overlapped extended segments form clusters; (**d**) interconnected clusters form a percolated network. The highlighted trapped segments can store elastic energy. The matrix can also contain individual supramolecular bonds, binary associations, or even multiplets. Reprinted with permission from [45], Copyright 2021, Royal Society of Chemistry.

In the case of ionomers, supramolecular units mainly consist of clusters representing domains of irregular aggregates [13,32,33,34,35,36]. The formation of clusters in both ordered and irregular morphologies can affect many important properties of polymers. Understanding the mechanism of formation of such clusters makes it possible, if necessary, to deliberately influence the regularity of their structure. The basic principles of the initial stage of phase separation are the same for both clusters of irregular aggregates and ordered hierarchical assemblies [37,38,39,40,41,42,43,44]. Regardless of the type, non-covalently associated units in supramolecular polymeric materials follow specific reaction pathways with the formation of large assemblies, comparable to the mechanisms of synthesis of covalent polymers. A schematic representation of the phase separation process in supramolecular polymeric materials is shown in Figure 1 [45]. The phase separation process consists of several successive stages, including (a) overlapping of functionalized regions of adjacent chains with the primary doublet connection; then (b) sequential association of doublets into quadruplets, sextuplets, and multiplets; in the next stage (c), multiplets overlap into clusters, an important feature of which includes the ability to exhibit their own glass transition temperature; and finally (d) the possibility of cluster merging appears, which cannot always be realized due to the intertwining of adjacent chains. In the case where adjacent clusters nevertheless link, a percolation network arises in the polymer matrix of ionomers, in which ionic aggregates perform the function of physical cross-linking. As a result, the mobility of macromolecular chains decreases, and the thermomechanical and rheological properties of polymers undergo significant changes.

Thus, an important feature of ionic groups is their tendency to sequentially combine into multiplets, and then into cluster supramolecular ionic formations [46,47,48,49,50,51]. This results in thermally reversible microphase separation of clusters and the non-polar polymer phase. The reversibility of microphase separation and the underlying processes of intermolecular interactions and reversible crosslinks determine the main properties and areas of application of ionomers. The hierarchical formation of the supramolecular structure of ionomers has a significant impact on the dynamics and physical and mechanical properties of materials. Through the engineering of secondary structures, it becomes possible to create and control the degree of hierarchy of structures, which makes it possible to change and regulate the physical and mechanical properties of ionomers. The continuous decrease in overall entropy accompanying chain growth leads to the aggregation of oppositely charged ionic groups into clusters, which, due to differences in the conditions of their formation, exhibit a wide size distribution. The difference in cluster size in turn determines differences in their strength and the characteristics of relaxation transitions [52,53,54,55,56]. Although the clusters are usually in the amorphous phase, high cluster concentrations increase the rigidity of polymers and decrease their crystallinity [57,58]. Electrostatically interacting groups covalently bonded to the polymer backbone can improve mechanical strength [59,60].

Ionic groups covalently attached to the ionomer backbone are commonly referred to as pendant ions. Free ions that electrostatically neutralize pendant ions are counterions. The cluster sizes are influenced by the ionic density and the nature of counterions. Changes in the nature of counterions lead to macroscopic changes in the properties of the ionomer [61,62,63,64,65]. For example, depending on the nature of the cation (sodium or potassium) used to neutralize the anions in Surlyn^®^, the degree of crystallinity, rheological properties and mechanical characteristics change due to a shift in the equilibrium between free ions and ionic clusters.

The ionic functions of ionomers are determined primarily by the type of ions (cations or anions) that serve as the basis for their functionalization. Ionomers containing anionic pendant ions are generally referred to as ***anionic ionomers***, while ionomers containing cationic pendant ions are referred to as ***cationic ionomers***. Sulfonate, carboxylate, acrylate, phosphoric and phosphonic acid groups are used to develop anionic ionomers [46,66,67,68,69,70,71,72,73]. Neutralization of anionic pendant ions can be accomplished using monovalent counterions such as alkali metal ions and organic alkyl ammonium ions. In the case of anionic ionomers, alkaline earth ions and transition metal ions, for example, can be used as multivalent counterions.

Some of the most representative options for cationic ionomers are polymers with imidazolium, quaternary ammonium, and pyridinium functional groups. Multivalent pendant cations are also known (e.g., 1,4-diazabicyclo[2.2.2]octane (DABCO) double ammonium salt) [74,75,76,77,78,79,80,81,82,83,84,85,86,87,88,89,90,91,92]. Monovalent pendant ions such as halides and tosylate anions are used to neutralize monovalent pendant cations. Neutralization of anionic pendant ions can be carried out using either monovalent counterions such as the alkali metal ions, organic alkyl ammonium ions, or multivalent counterions such as the alkaline earth ions, transition metal ions. Dipole–dipole interactions involving ion pairs lead to the formation of a physical polymer network. Multivalent counterions, capable of forming ionic bonds between two monovalent pendant cations, offer a wider range of Coulomb interactions and the potential for forming stronger physical crosslinks. In the development of ionomers, more and more work is currently being directed toward the use of multivalent counterions.

### Application of Ionomers

The presence of ionic interactions and their reversible clustering cause sensitivity to stimuli and the emergence of energy dissipation capacity [93,94,95,96,97,98], promote polymer miscibility and facilitate ion transport [99], resulting in high ionic conductivity [100,101,102,103,104,105,106]. Clustering of ionic groups also contributes to the strengthening of ionomers [107].

Using ionomers, it was possible to develop actuators whose key practical application lies in the polymers’ ability to convert electrical, pneumatic, or hydraulic input energy into controlled mechanical force. The ability to subsequently actuate robotic arms, valves, and lifting mechanisms connected to the ionomer actuators via sensors makes it possible to set them in motion [67,102,108,109].

Ionomers can be used to produce shape-memory materials [48,49,110,111,112,113,114,115,116,117,118,119,120,121], which are characterized by the ability to return to a given shape after deformation, and self-healing materials [122,123,124,125,126,127,128,129,130,131,132,133,134,135,136,137,138], which are capable of self-healing by contracting cracks after damage. Shape-memory polymers are used in areas such as biomedical devices, electronics, and the aerospace industry.

Ion-exchange resins made from ionomers are capable of selectively exchanging ions in solution and are therefore used for water treatment and purification [103,139,140]. This process removes heavy metals and softens water by replacing calcium and magnesium ions with sodium or potassium ions. Because ionomer resins are stable and durable, they are suitable for repeated use in industrial and domestic water treatment systems.

Ionomers are ideal materials for use in proton exchange membranes in fuel cells [7,8,9,66,72,78,91,99,104,105]. Critical to the efficiency of these devices is their ability to selectively transport ions, allowing energy to be generated through controlled chemical reactions.

Water purification membranes based on ionomers are able to pass clean water and thus selectively remove contaminants, allowing for safe and efficient filtration processes [68,141,142].

Ionomers can exhibit high adhesive properties and resistance to environmental influences, making surfaces more durable. Being resistant to chemical attack, abrasion, and corrosion, ionomers are capable of creating smooth, uniform protective layers. This makes them suitable for applications as coatings and paints in the automotive industry and in objects requiring both aesthetic and functional surface protection [75,143,144,145]. Since ionomers have self-healing properties, heat treatment can eliminate minor scratches, extending the service life of products.

High adhesive strength to metals, plastics, and glass, combined with flexibility, makes ionomers suitable for use in the automotive, construction, and consumer goods industries as adhesives and sealants [146,147,148]. Importantly, ionomers maintain flexibility under conditions where materials expand or are subjected to mechanical stress. Excellent resistance to environmental conditions such as temperature fluctuations and moisture ensures a long service life even in harsh climates.

Ionomers can combine strength, puncture resistance, and tear resistance with optical clarity, sealing properties, and heat sealability. The combination of these properties, along with resistance to oils and fats [2,3,4,5,6,7], makes them ideal materials for creating food packaging films.

Ionomers can conduct ions and create an insulating barrier for electrons [63,64,92], making them promising for use in electrochemical devices such as batteries. The stability of ionomers ensures the efficiency and long-term performance of these devices.

## 2. Anionic Ionomers

### 2.1. Sulfonic Acid-Based Ionomers

The best known is the perfluorinated sulfonic acid ionomer (PFSI), which has found application as the proton exchange membranes (PEMs), catalyst system binders in fuel cells, water electrolyzers, and carbon dioxide reduction devices. The most widely used PFSI is Nafion, which consists of a perfluorocarbon backbone copolymerized with a strongly acidic sulfonic acid side chain [149,150,151,152,153,154,155,156,157,158,159,160,161,162,163]. The supramolecular structure of the Nafion is based on a hydrophobic, semicrystalline matrix in which phase-separated morphology is constructed by the hydrophilic sulfonate water channels [164]. The exact structure of the water channels is still being debated, but the most common idea is that they are cylindrical [151,152,165,166,167,168,169,170]. In ref. [171], the influence of the physicochemical properties of the fluoropolymer matrix on the formation of the supramolecular structure and ionic conductivity in perfluorinated sulfonic acid ionomer thin films was studied. In situ water sorption experiments were conducted for polymers, during the synthesis of which the ratio of Nafion and a perfluorodioxolane ionomer with the same sulfonic acid side chain and mass fractions was systematically varied. In dry form, perfluorinated sulfonic acid is in undissociated or protonated form with a very low water content, one molecule of water per sulfonic acid. It was suggested that the occurrence of ionic conductivity and morphological rearrangement are caused by the same physical process. According to small-angle X-ray scattering measurements of the grazing incidence, ionomer film materials rapidly form ionomer domains when interacting with water, but their subsequent ordering occurs at a significantly slower rate. Experiments have led to the conclusion that thin ionomer films are characterized by slow segregation of ionomer domains during hydration, which provide proton conductivity. High segmental mobility of fluoropolymer matrices is necessary to increase the efficiency of coalescence processes in ionomer structures.

**Figure 2:**
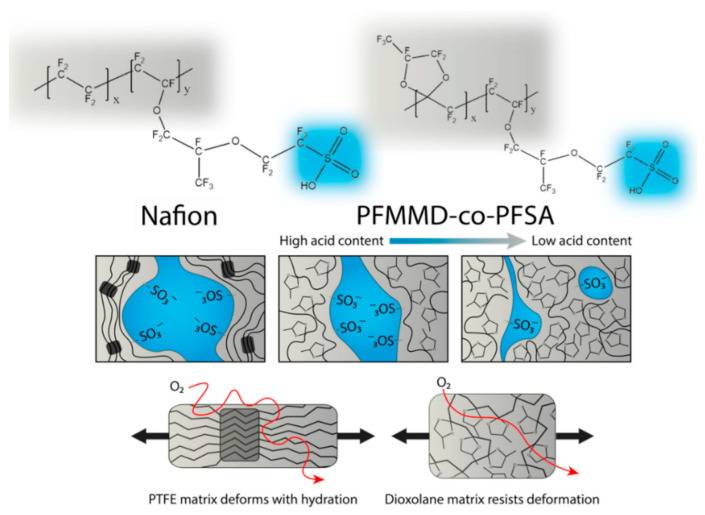
PFSIs such as Nafion (top left) typically comprise a PTFE backbone and a sulfonic acid side chain. Polarity differences in these groups drive phase separation into acid-rich ionomer domains in a semicrystalline hydrophobic matrix with the size and shape of ionomer domains dictated by the interplay between ionic strength, which drives water sorption, and mechanical deformation of the fluoropolymer matrix. Emerging ionomers such as PFMMD-co-PFSA (top right) introduce bulky dioxolane groups into the polymer matrix, altering the phase separation behavior. In this study, we explore the role of matrix physicochemical properties and ionomer ionic strength in the evolution of transport properties and structure morphology of ionomer domains. Reprinted with permission from [171], Copyright 2020, American Chemical Society.

A schematic of the supramolecular organization of the Nafion, in which the phases are separated into acid-rich ion-containing domains embedded in a hydrophobic fluoropolymer matrix, is shown in Figure 2. Study [171] found that the backbone of the PFSIs consists of poly(tetrafluoroethylene) (PTFE) with a sulfonic acid side chain attached. The size and shape of the ionomer domains is the main element determining the efficiency of water sorption, and ionic strength interactions influence the mechanical properties of the fluoropolymer matrix. Dioxolane groups are introduced into the polymer matrix of poly[perfluoro(2-methylene-4-methyl-1,3-dioxolane)-co-perfluoro(4-methyl-3,6-dioaoct-7-ene)sulfonic acid] (PFMMD-co-PFSA) studied by the authors. These groups alter the nature of the supramolecular organization, the physicochemical properties of the matrix, and the ionic strength of the ionomer. The relationship between the structural morphology of ionomer domains and the transport properties of the resulting membrane materials was studied.

**Figure 3:**
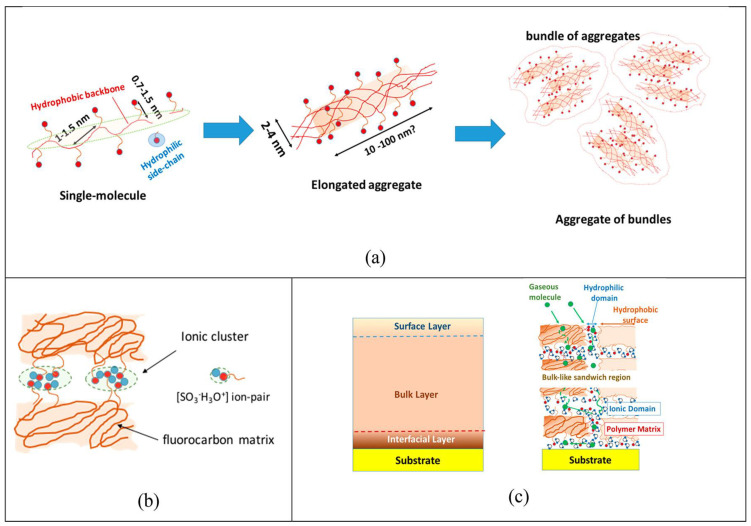
Hierarchical structure of the PFSA ionomer: comblike architecture of an isolated extended PFSA ionomer molecule, its elongated nanoaggregated structure in hydroalcoholic media, and bundles of nanoaggregates (**a**). Clustering of ion pairs within a hydrophilic domain. (**c**) Depiction of the three-layered structure of a polymer thin film and an ionomer thin film showing phase-segregated morphology (polymer matrix and hydrophilic ionic domain), a water-rich interfacial region, and a parallel pathway for gaseous molecule transport (**b**). Reprinted with permission from [172], Copyright 2019, American Chemical Society.

In ref. [172], the supramolecular organization in perfluorinated ionomer films is described as follows. Figure 3a initially shows an isolated elongated comb-shaped molecule of the perfluorinated sulfonic acid. At higher concentrations in dispersions, ionomer nanoaggregates combine together to form a weakly aggregated structure. Then, its nanoaggregated structure in aqueous-alcoholic media and bundles of nanoaggregates are shown. In the dry solid state, sulfonic acid is in the undissociated or protonated form with a very low water content: one water molecule per sulfone group. Strong electrostatic interactions lead to clustering of the sulfone groups, thereby cross-linking these polymers with low-surface-energy fluorocarbon backbones (Figure 3b). These ion clusters are hydrophilic in nature, form a separate phase of viscoelastic material within the hydrophobic Teflon matrix, and control many different behavioral characteristics of ionomers. Ionic interactions in these clusters also create an additional relaxation mode of the polymer, and hydrated ion clusters control key transport properties of the ionomer. When exposed to a humid environment, hydrophilic ion clusters/domains/channels are filled with water, providing a pathway for the transport of ions, liquid water, and dissolved gaseous molecules (Figure 3c). The authors of [172] showed that when ionomers are embedded in nanothin films, a wide range of structural organization is expected, providing a rich variety of surface, interfacial, and bulk characteristics. In self-assembly of ionomers, the dispersion medium and the hydrophobicity/hydrophilicity of the substrate can lead to partial or even no coverage of the substrates, shedding light on the complexity of polymer-substrate, polymer-solvent, and polymer–polymer interactions, an understanding completely obscured when using the spin-coating method to create films. The authors demonstrate that the same ionomer can be used to create a variety of surfaces, from superhydrophilic to highly hydrophobic, by controlling the film thickness or by choosing the substrate material. Ultrathin hydrophilic surfaces self-assembled ionomer Nafion films exhibit wettability switching behavior, which opens the possibility of creating smart surfaces responsive to external stimuli. The thermally responsive behavior of the films is discussed in the context of surface (wettability) and bulk (thermal expansion) characteristics, as well as a recently discovered vibrational mode. Thermal expansion coefficients dependent on substrate and film thickness confirm the importance of interfacial interactions and the limitations of the structure and properties of these films. They also open the possibility of tuning the bulk properties of the ionomer through substrate chemistry. A systematic analysis of factors influencing proton conductivity is presented to elucidate the unresolved causes of the suppressed conductivity of nanothin ionomers compared to the bulk membrane.

The goal of researchers is to create cheaper membranes that could function at lower humidity and higher temperatures by improving the percolation hydrated domains [173,174,175,176,177,178]. Most of the ionomers described in these studies are amorphous, and their morphology, unlike Nafion, is poorly controlled. As a result, the performance of Nafion is not achieved for these polymers. Highly ordered morphology, which promises the development of new membranes for the efficient transport of protons, ions, and even small molecules, can be achieved according to [179,180] by using the strategy of controlled hairpin polymer folding. There are examples of ionic conductivity in polymers with crystal-like ordering. By precise control of the chain microstructure via acyclic diene metathesis synthesis, it became possible to tune chain folding and chain morphology. In ref. [181], controlled chain folding and layers of acid groups were achieved through the periodic placement of pendant atactic carboxyl groups along linear polyethylene. In the work of the same scientific group [182], carboxyl groups were replaced by sulfonic acid groups and, as a result, hydrated layers with high proton conductivity were formed.

In ref. [183], it was established that by precisely placing the -SO_3_H groups on every 21st carbon atom, it becomes possible to form hairpin chain folds at the position of each sulfonic acid group. Controlled folding of the polyethylene and self-assembly into a crystalline structure, triggered in this way, provide the desired proton conductivity. In the same work, it was shown that anisotropic conductivity ensures the alignment of the layers such that the layer normal is orthogonal to the desired transport direction. Alignment can be initiated by mechanical shear, exposure to an electric or magnetic field, or using directional epitaxial solidification.

**Figure 4:**
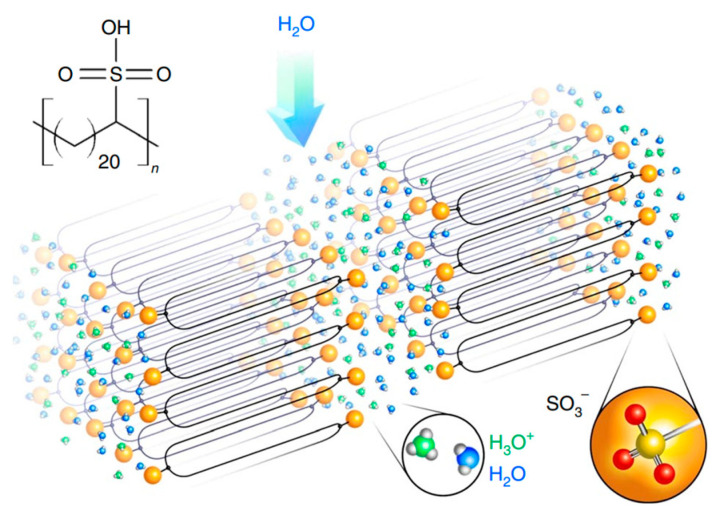
Chemical structure and schematic rendering of the secondary structure of hydrated p21SA, showing the crystalline backbones and acid-lined water layers. At bottom right, the yellow atom is sulfur and the red atoms are oxygen. Reprinted with permission from [183], Copyright 2018, Nature Portfolio.

The calculations carried out allowed the authors to establish that the high degree of ordering of water domains in precisely sulfonated polyethylene improves proton conductivity relative to random tortuous water channels. High cationic conductivity can be achieved by concentrating acid groups within the layers. Since the proximity of acid groups can ensure efficient ion transport, conductivity becomes possible even in the absence of water. Figure 4 shows the chemical structure, schematic rendering of the secondary structure of hydrated precisely sulfonated polyethylene, the crystalline backbones and acid-lined water layers.

**Figure 5:**
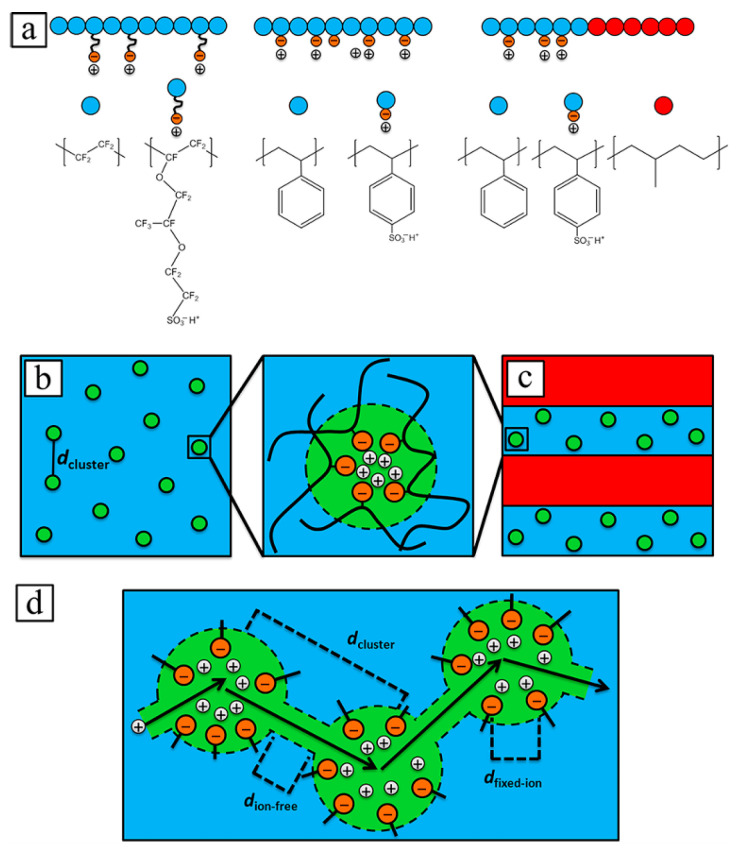
(**a**) Examples of molecular structures for different types of ionomers, from left to right: a random copolymer Nafion, a random copolymer sulfonated polystyrene (PSS), and a block copolymer sulfonated polystyrene-block-polymethylbutylene (PSS-PMB). Schematics of ion clusters in (**b**) random ionomers such as PSS and Nafion and (**c**) a block copolymer. The green spheres represent the ionic clusters that are a distance, d_cluster_, apart. (**d**) Proton transport through a membrane containing hydrated ionic clusters a distance d_cluster_ apart, with a region free of ions between the clusters a distance d_ion-free_ long, with fixed charges inside the clusters being on average a distance d_fixed-ion_ apart. Reprinted with permission from [184], Copyright 2012, American Chemical Society.

In [184], the effect of clustering on proton transport in PEMs was investigated. It was suggested that proton transport is hindered by the presence of clusters. The study also considered an approach to obtaining PEMs in the absence of clusters. Figure 5a shows the structures of the most interesting polymers, the simplest of which is sulfonated polystyrene (PSS). Figure 5a also shows the structure of Nafion and the structure of a block copolymer (PSS-PMB) containing a PSS block and an uncharged polymethylbutylene (PMB) block. Figure 5b shows clusters formed in Nafion and PSS. Figure 5c illustrates a schematic representation of ionic aggregates formed in PSS-PMB, in which clustering is limited to one of the microphases. The spherical morphology of ionic clusters was first proposed for PSS in [185], and for Nafion in [186]. As mentioned above, proton-ion membranes in fuel cells operate only in a hydrated state. Under low water conditions, the ion clusters swell, but as the water content increases, a percolation network of hydrated channels forms between the clusters (Figure 5d). One might expect the percolation channel to be devoid of clusters; however, instrumental studies have shown that clusters are present in both dry and hydrated samples.

**Figure 6:**
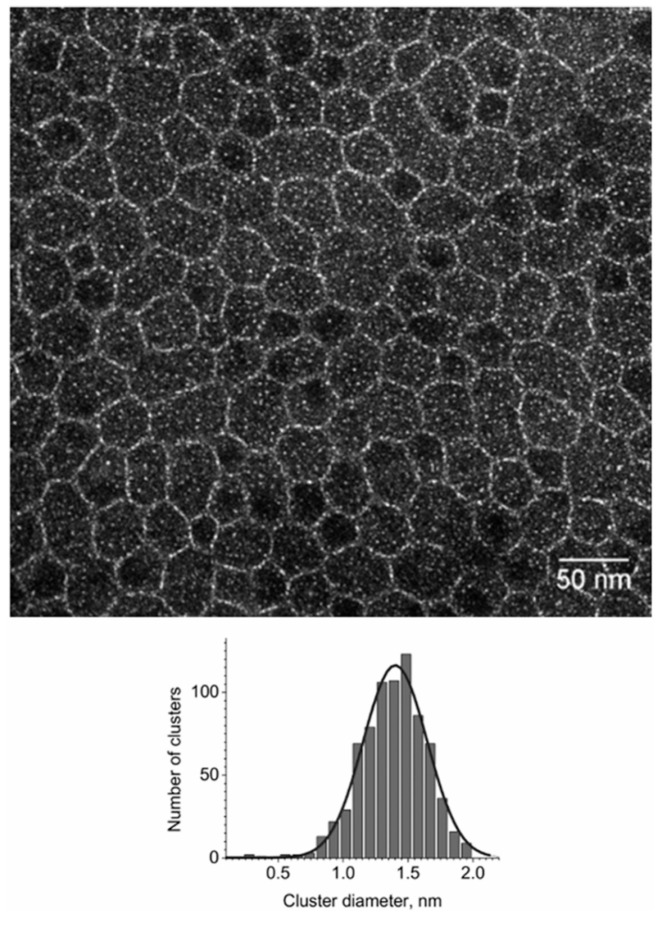
PSS − PMB film with a thickness of 35 nm prepared by casting and annealing in water vapor, showing the honeycomb morphology and ionic clusters (bright spots). Histogram of ionic cluster size distribution (bars) and Gaussian fit (curve). Reprinted with permission from [187], Copyright 2011, American Chemical Society.

Rheology, electron microscopy, and scattering are used to study clusters. According to study [29,31], the rheological properties of ionomers are similar to those of chemically cross-linked polymers. Due to the small size of the clusters, the limited stability of polymers in the electron beam, and the lack of contrast between the clusters and the hydrophobic matrix, it has proven difficult to obtain direct evidence of the presence of clusters using electron microscopy. A clear micrograph of acidic clusters in polymer electrolyte membranes (Figure 6) is presented in [187], which may be one of the few available to date.

Some of the common electrolytes are solutions of metal salts in polyethylene oxide (PEO). The mobility of ions in polymer electrolytes is closely related to the segmental motion of the polymer chain [188,189]. However, in the conductors obtained in this way, undesirable accumulation of anions occurs at the interface between the electrode and the electrolyte. Ultimately, the effective field on the cations decreases and the applied field required for cation transport increases. Due to the formation of ion clusters, the ionic conductivity in ionomers with alkali metal cations is usually significantly lower than in solutions of metal salts in polyethylene oxide.

**Figure 7:**
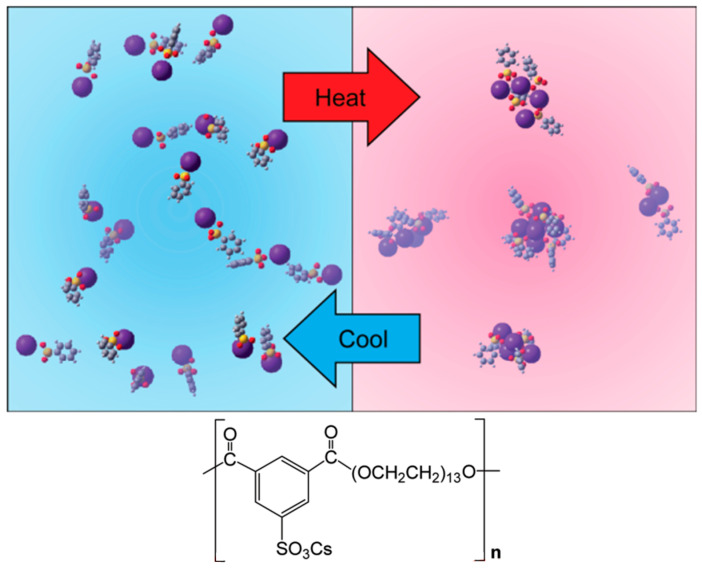
Schematic showing the SO_3_^−^ Cs^+^ ion pairs in PEOx-100% Cs ionomers that reversibly transform between isolated ion pairs and ionic aggregates. At low temperature the isolated ion pairs have an average correlation distance between ion pairs of ∼0.9 nm (X-ray scattering maximum at ∼7 nm^−1^). At elevated temperatures the SO_3_^−^ Cs^+^ ion pairs aggregate to form traditional ionic aggregates with an interaggregate separation of ∼2.1 nm (X-ray scattering maximum at ∼3.0 nm^−1^). Reprinted with permission from [190], Copyright 2011, American Chemical Society.

In [190], a series of sulfonate polyether ionomers with clearly defined lengths of PEO spacers between phthalates was developed. Alkali metal cations were used as counterions. Ionic conductivity in these systems is determined by the polymer mobility and the state of ion pair aggregation, which increases with increasing temperature and with increasing cation size. Thus, in ionomers where cesium is used as a counterion, isolated ion pairs transform into ionic aggregates below 40 °C at 120 °C (Figure 7). Moreover, the length of the polyethylene oxide spacer has little effect on the degree of ionic aggregation. The temperature dependence of the concentration of conducting ions in these ionomers deviates from the dependence in Arrhenius coordinates due to the possibility of the existence of several activation energies. This may be due to a change in the behavior of ion associates with increasing temperature.

Some of the most studied polyester ionomers are sulfonated poly(ethylene terephthalate) ionomers (SPET) in the Na^+^ form. The advantage of fibers obtained from them is the possibility of achieving significantly higher dyeing efficiency [191,192]. Nevertheless, strong ionic interactions arising in such ionomers slow down the rate of crystallization, increase melt viscosity and thus significantly impair processability [193,194,195,196]. SPET are also used to compatibilize polyester-polyamide blends due to the high affinity between sulfonate salt functionalities and the amide units of the polyamide [197,198,199,200,201].

**Figure 8:**
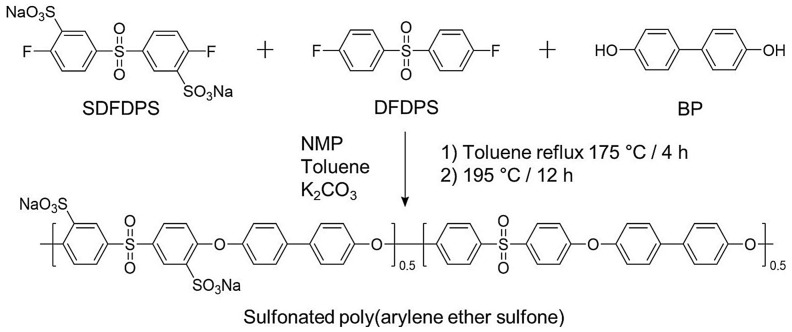
Synthetic scheme of sulfonated poly(arylene ether sulfone) copolymer (SP50, degree of sulfonation = 50 mol%). Reprinted with permission from [202], Copyright 2025, Elsevier B.V.

In [202], a five-layer composite membrane incorporating a radical scavenger was successfully fabricated using poly(arylene ether sulfone) ionomer with a degree of sulfonation of 50% (Figure 8), polytetrafluoroethylene substrates, and CeO_2_ nanoparticles. The composite membrane exhibited excellent physical and chemical stability, dimensional stability, and mechanical properties due to the porous PTFE and the radical scavenger, which was cesium oxide.

The authors of [203] focused on the rheological behavior of sulfonated polystyrene, showing that the addition of metal cations shifts the equilibrium in favor of cluster ion pairs, which, in turn, increases the rigidity and viscosity of the melt.

**Figure 9:**
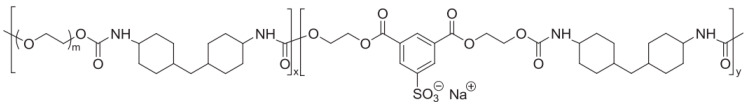
Synthesis of sulfonated polyurethanes bearing sulfonated hard segments (SHSPU). Reprinted with permission from [204], Copyright 2012, Elsevier B.V.

In [204], a polyurethane sulfone ionomer was successfully synthesized based on polyethylene glycol using a sulfonated monomer. Sulfonate anions were introduced into the polyurethane backbone during urethane formation (Figure 9). Sulfonation of already synthesized linear polyurethanes is also widely used to obtain sulfonated polyurethanes. This method of introducing sulfonate anions allows for easy regulation of the number of ionic groups.

One possible approach to developing new energy storage devices is to use latent heat storage materials (PCMs). This technology utilizes the latent heat of fusion, which occurs at the phase transition temperature of the PCM, and provides high heat storage density and heat retention capacity. Solid–solid PCMs are particularly suitable because they do not form liquid or gaseous products, the volume of the materials changes little during phase transitions, and sealing is not required. Polyurethanes produced using poly(ethylene glycol) (PEG-PU) as a soft segment and phase transition ingredient are promising in this regard [205,206,207,208,209,210].

In the PEG-PU structure, poly(ethylene glycol) is linked to a diisocyanate, which functions as a hard segment and the backbone of the polymer matrix, maintaining the material in a solid state after melting the PEG. To increase the enthalpy of the phase transition of poly(ethylene glycol) in PEG-PU, it is necessary to increase the degree of crystallinity of PEG. This is hindered in the PEG-PU matrix by the hard segments, which interfere with the growth of PEG crystals as a result of their dispersion in soft domains [211,212].

The interaction between the hard and soft segments through hydrogen bonding, characteristic of polyurethanes, also contributes to the restriction of the free movement of poly(ethylene glycol) and, accordingly, to the processes of agglomeration and crystallization of PEG. As has been shown in [213,214], the introduction of ions into polyurethanes can lead to a weakening of the interaction of the domains of the hard and flexible segments and, accordingly, to an increase in the processes of their segregation [215,216]. The creation of ionogenic groups in polyurethanes can also lead to freer movement of poly(ethylene glycol) macrochains and an increase in their degree of crystallization [217].

**Figure 10:**
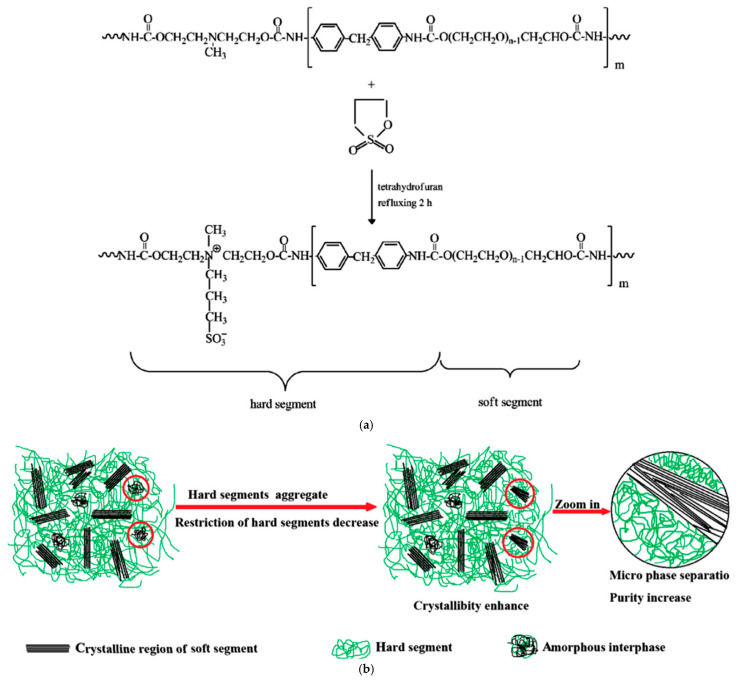
Synthetic route for PU ionomers (**a**) schematic illustration of ionic groups effect on the increment of the crystallinity and the enthalpy of PU ionomers (**b**). Reprinted with permission from [218]. Copyright 2014, Elsevier B.V.

In the work [218] linear polyurethane ionomer PCMs were synthesized using PEG, 4,4-diphenylmethane diisocyanate, N-methyldiethanolamine and 1,3-propanesulfonate (Figure 10). The results showed that PU ionomers have high heat-accumulating capacity, and the phase transition enthalpies of ionomer polyurethanes were 142.5–152.3 J/g. The introduction of polar ionic groups leads to an increase in the crystallization capacity of soft poly(ethylene glycol) segments, the phase transition enthalpy of ionomers and the thermal stability of ionomer PUs. It is important to maintain the polymers in the solid state at a temperature above the melting point of the poly(ethylene glycol) phase.

### 2.2. Carboxylic Acid-Based Ionomers

The production of ionomeric cluster structures is of clear interest in the development of elastomers capable of self-healing at ambient temperatures [219,220,221]. A distinctive feature of such ionomeric elastomers is their ability to recover from cuts, which occurs due to the rupture of dynamic ionic bonds upon cutting. Upon contact between the cuts, the flexible polymer chains self-diffuse and reform broken crosslinks, restoring the integrity of the sample at ambient temperatures. Dynamic crosslinks formed through ion–ion interactions can impart high tensile strength to elastomers. Nevertheless, designing dynamic crosslinks with the desired strength and service life is a complex task [222,223,224,225,226].

A methodology for regulating the strength and service life of dynamic crosslinks in ionically crosslinked polyisoprene (PI) was developed in [227]. Ionic fragments in PI were shown to undergo so-called “ionic hopping,” during which they continuously move between ionic clusters, and the elastomer exhibits a strong and elastic response to rapid stretching. Under slow stretching, PI can exhibit superelastic properties. It was found that the hopping rate of ionic fragments can be controlled by the level of their neutralization. Due to the resulting dynamic ionic crosslinks, PI spontaneously self-heals without the use of heat.

**Figure 11:**
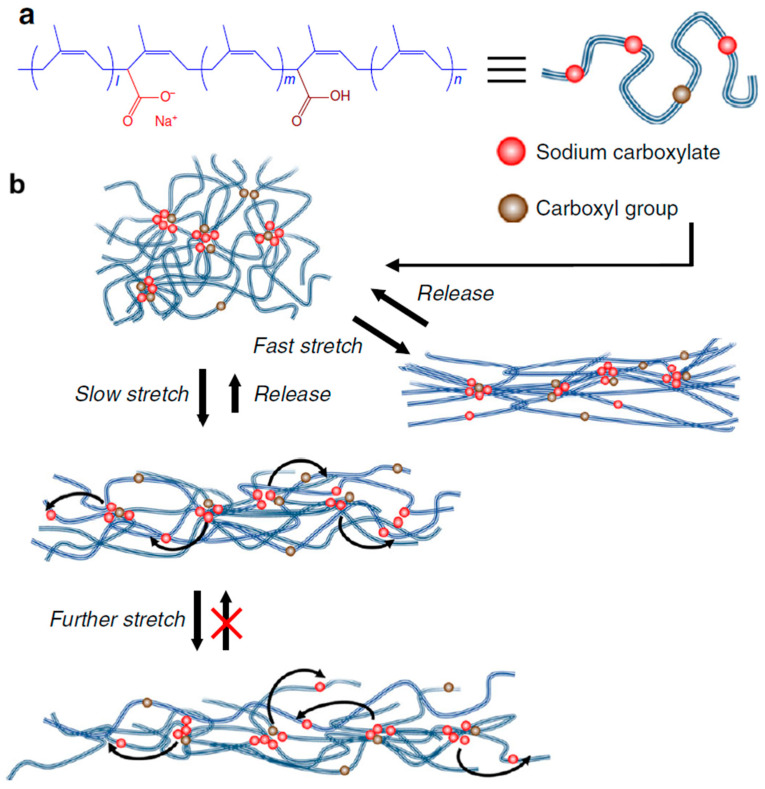
Chemical structure of PI–xNA and a schematic of its behavior under stretching (**a**) Chemical structure and schematic illustration of PI–xNa. Schematic description of proposed mechanisms for elastic response under rapid stretching and viscoelastic deformation under slow stretching. Under fast stretching, ionic aggregates act as strong crosslinks. Conversely, when stretching is slow, ionic groups attached at stressed polymer chains detach from ionic aggregates and enter other aggregates to dissipate the loaded force (**b**). Reprinted with permission from [227], Copyright 2018, Nature Portfolio.

According to the scheme shown in Figure 11, carboxyl groups were bound to 1.7% of the *cis*-1,4-polyisoprene monomer units. Between 24 and 90% of the attached carboxyl groups were neutralized using sodium ions. As a result, the inclusion of a small proportion of ionic groups in the inherently hydrophobic PI leads to the formation of ionic aggregates that act as physical crosslinks.

Thus, improving the mechanical behavior of ionomers depends both on the properties of the polymer matrix and on the nature of the ions and counterions. In the case of elastomeric ionomers [228,229,230,231,232,233,234], ionic crosslinks effectively improve strength due to their ability to reversibly dissociate. Nevertheless, the existence of ionic interactions, including in the form of their aggregates, can only rarely make an effective contribution to strengthening mechanisms [235,236,237]. In contrast, in the case of glassy polymers, ionic clusters can lead to the production of hard and brittle polymers, and the development of a general approach to the molecular architecture for their strengthening through ionic interactions remains a complex task.

To weaken electrostatic interactions, shorten the association lifetime of ionic motifs in the ionomer network and, accordingly, increase the ion mobility, it is necessary to use larger ions [1,238,239,240,241]. It was shown that during the deformation of sulfonated polystyrene ionomer samples, the transition from a brittle to a plastic state is accompanied by continuous dissociation and reassociation of ionic bonds. The association time depends on the size of the alkali metal ions used as counterions. However, to be able to control the counterion sizes, it is promising to use bulky organic cation-active molecules. In this case, it becomes possible to influence the ability to screen the charge by using alkyl chains of varying lengths directly linked to cation-active groups. The simultaneous manifestation of both Coulombic and elastic forces in glassy polymers causes competition between the ion diffusion activation energy and ion size, which is nonmonotonic in nature [242]. Factors such as spatial confinement and the effect of local concentration on polymer dynamics, and the dissociation constants of ionogenic groups in ionomers have significant differences compared to their low molecular weight analogs [243,244,245,246,247,248].

**Figure 12:**
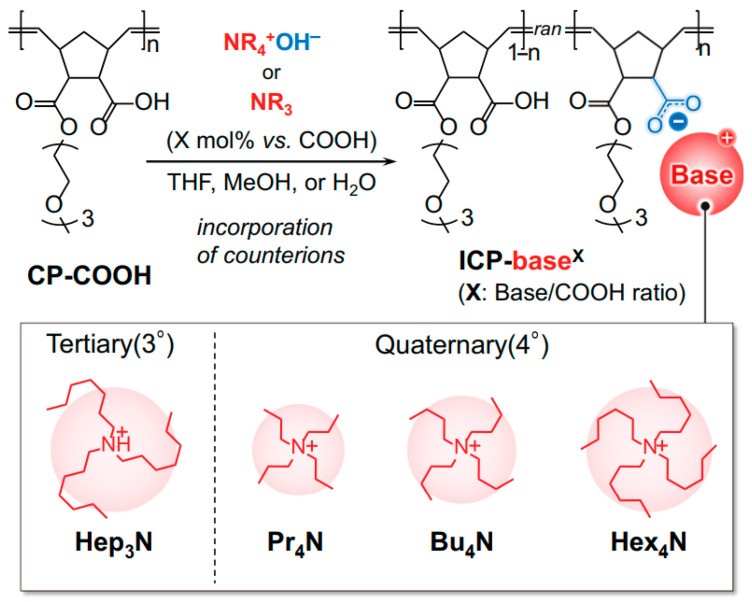
Chemical structures of CP-COOH and ICP-base^X^. Reprinted with permission from [249], Copyright 2025, Nature Portfolio.

Because of the complexity of the interactions between ion mobility, backbone rigidity, and plasticizing effects, understanding the influence of organic counterions on the mechanical behavior of glassy polymers requires further research. In [249], the influence of tertiary and quaternary ammonium counterions on the mechanical properties of ionic comb polymers was studied (Figure 12). The researchers’ attention was directed to establishing the influence of the counterion incorporation ratio and the size of the ionic quaternary ammonium counterion on the mechanical properties of polymers consisting of polynorbornene with triethylene glycol monomethyl ether side chains and carboxylic acid groups in each monomer. A range was found in which, due to a combination of ionic crosslinking and plasticization effects caused by the presence of partially neutral amines, only tertiary ammonium counterions, and not quaternary ammonium counterions, improved the mechanical properties of the polymers.

**Figure 13:**
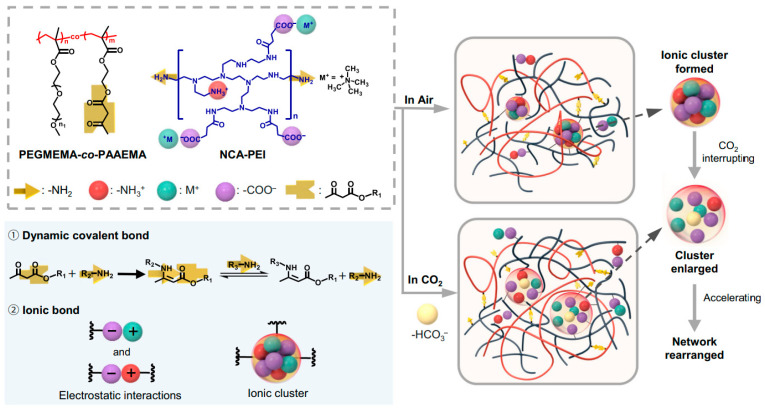
Schematic of the synthesis process of IC-CAN. Chemical structures (top-left panel) and cross-links (bottom-left panel) between PEGMEMA-co-PAAEMA and NCA-PEI, and illustration of CO_2_-triggered molecular rearrangement in the IC-CAN (right panel). Reprinted with permission from [250], Copyright 2024, Nature Portfolio.

The study [250] is the first reported covalent adaptable network incorporated with ionic clusters (IC CAN). The polymer was synthesized by reacting neutralized carboxylic acid modified branched poly(ethyleneimine) (NCA-PEI) ionomer with statistical copolymers of poly(ethylene glycol) methyl ether methacrylate and 2-acetoacetoxyethyl methacrylate (PEGMEMA-*co*-PAAEMA). The cross-linking process of the NCA-PEI with PEGMEMA-*co*-PAAEMA is driven by combining the dynamic covalent interactions, i.e., vinylogous urethane bonds, and the ionic interactions between the carboxylate and amine groups, as shown in Figure 13. With strong electrostatic interactions among –NH^3+^, –COO^−^, and N^+^(CH_3_)_4_ (shortly named M^+^) in a matrix with a relatively low dielectric constant, ions have a strong tendency to pair or form larger aggregates [251].

Clustering occurs due to the overlap of dense aggregates surrounded by molecules with limited mobility [252]. The loosening of ionic clusters with increased ion mobility is influenced by the interaction of carbon dioxide with amine organobases. As a result, segmental mobility increases, the dynamic process accelerates, and topological rearrangement of the cross-linking networks occurs. The combination of dynamic covalent bonds and CO_2_-sensitive ionic interactions enables the recycling of the IC CANs under the influence of carbon dioxide.

Shape memory polymers (SMPs) can change shape under the influence of an external stimulus. This can be the effect of temperature, the presence of certain ions, light radiation, changes in the acidity of the environment, electric or magnetic fields [253] have a permanent shape. Due to the existence in thermally activated SMPs of both a crosslinked network and a second, reversible network, such polymers can be deformed after heating and remain in the new acquired shape. However, this shape is temporary and can exist only until reheating above the glass transition temperature of a second, reversible network. In the absence of external stress, the material is restored. Thermal SMPs are promising for use in sensors, mechanical drives and medical devices [48,254,255]. To develop a polymer with a shape memory effect, crystals obtained on the basis of an ionomer and a fatty acid or fatty acid salt (FAS) were used in [256,257]. The existence of a strong supramolecular bond between FAS and an ionomer motivated the development of an elastomeric ionomer, the zinc salt of sulfonated poly(ethylene-co-propylene)-*co*-(5-ethylidene-2-*nor*bornene), and a high-temperature thermoplastic SMP from sulfonated poly(ether ether ketone) [258,259].

**Figure 14:**
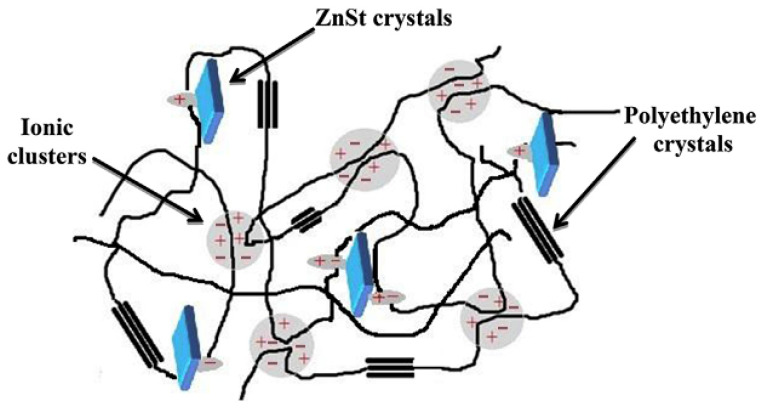
Schematic of the microstructure of PEMA(x > 0). Features are not drawn to scale. Reprinted with permission from [260], Copyright 2017, Elsevier B.V.

In this SMP design, strong intermolecular interactions between FAS crystals and the ionomer allow FAS microdomains to function as temporary crosslinks in these systems, enabling the polymers to withstand stress. Ionic associations within the ionomer can function as a permanent network, but the primary function of the ionomer is to facilitate dispersion and stabilize the FAS dispersion. This provides additional functionality to the polymer, which acquires the ability to develop strong intermolecular interactions. The work [260] describes the mechanical properties and shape memory behavior of composites obtained on the basis of zinc stearate (ZnSt) and an ionomer representing poly(ethylene-*co*-methacrylic acid) (PEMA) (Figure 14).

**Figure 15:**
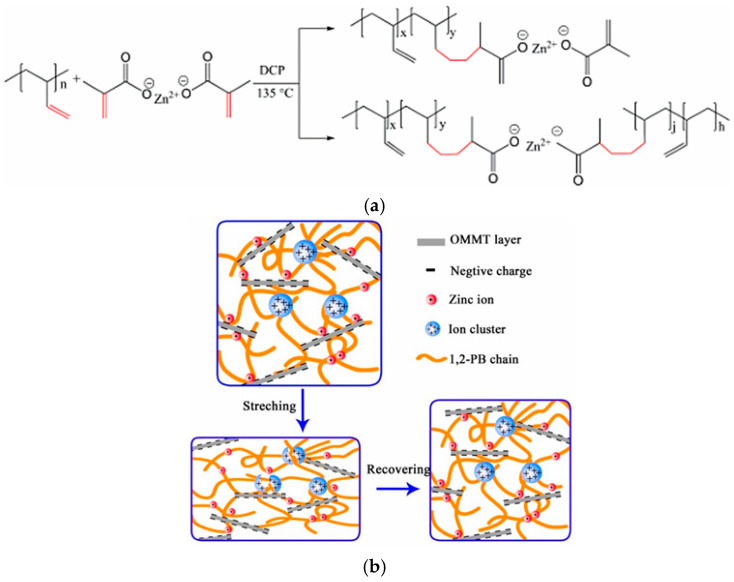
Reaction between 1,2-PB and zinc methacrylate (ZDMA) (**a**) Proposed mechanism of the rupture and reconstruction of sacrificial bonds in the multi-network system of 1,2-PB (**b**). Reprinted with permission from [261], Copyright 2019, MDPI.

The ionomer and ZnSt exhibit good compatibility up to 10 wt.% zinc stearate content. At ZnSt content greater than 10 wt.%, zinc stearate and ionomer partially miscibility, and improved mechanical properties of the resulting polymer are observed. At 30 wt.% zinc stearate content, a plastic material with a 50% higher tensile modulus was obtained. The shape memory effect was studied for a polymer obtained using an ionomer and 50 wt.% zinc stearate. In this case, ionic aggregates separated into nanophases served as the permanent network. The function of the reversible, temporary network was performed by the phase-separated ZnSt crystalline phase. Cyclic tests reproducing the shape memory effect were conducted over five consecutive cycles. The degree of shape retention was 92%, and its recovery reached 97%. In [261], a multi-ionic network was created in covalently cross-linked 1,2-polybutadiene (1,2-PB) by intercalating organic montmorillonite (OMMT) into the polymer in situ. Due to their dynamic nature, the ionic bonds formed in the composite are capable of rearranging and effectively dissipating energy, resulting in high strength and a low stress relaxation rate. The improved mechanical properties are a consequence of the ability of the ionic bonds formed in the composite to withstand and sacrifice the initial load, and then preferentially degrade before the rupture of the covalent network of elastomers. According to the scheme shown in Figure 15, zinc dimethacrylate (ZDMA) is grafted to 1,2-PB by interacting with the double bonds located in the 1,2-branches of polybutadiene. Dicumyl peroxide (DCP) was used as an initiator. It was found that as a result of the combination of -(COO)_2_Zn groups, ionic clusters are formed.

### 2.3. Phosphoric and Phosphonic Acid-Based Ionomers

Due to their high thermal and chemical stability, phosphonic acid-functionalized ionomers represent an alternative to sulfonated polymers as components of proton exchange membrane fuel cells [262,263,264]. The amphoteric nature and high permittivity of phosphonic acid groups allows self-dissociated acid groups to form hydrogen bond networks and creates the possibility of anhydrous proton conductivity at elevated temperatures [265,266,267].

Advances in the synthesis of phosphonated ionomers with precisely tuned arrangements of functional groups in the polymer backbone show that these materials allow significant control over the microstructure of ion clusters and thus clearly point the direction of future research [268,269]. However, due to the low acidity of phosphonic acid moieties, phosphonated polymers exhibit lower overall conductivity compared to their sulfonated analogs. This has necessitated the synthesis of polymers with a high content of phosphonic acid moieties. For example, in the works [270,271]. Highly phosphonated polymers with percolated ionic cluster networks, for which high proton concentrations were achieved, were obtained. Although the most desirably approach to obtaining such polymers was the polymerization of phosphonated monomers, which offers the advantage of a high (up to 100 mol%) level of phosphonation and predictable distances between phosphonic acid groups, their high tendency to aggregation has led to little research in this area [272,273,274,275].

**Figure 16:**
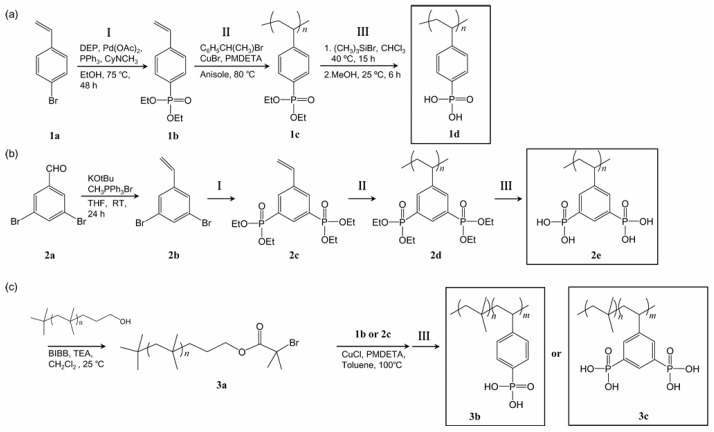
Syntheses of (**a**) polystyrene phosphonate (PSP); (**b**) polystyrene bisphosphonate (PSbP) homopolymers; (**c**) Poly(isobutylene)-b-PSP and Poly(isobutylene)-b-PSbP block copolymers Reprinted with permission from [279], Copyright 2018, American Chemical Society.

Therefore, another approach to the synthesis of phosphonated polymers was the postmodification of the polymer backbone [276] or side chains [277] with phosphonate moieties, allowing high phosphonation levels (up to 80 mol%) to be achieved [278]. In this case, the random nature of the phosphonation reaction does not allow precise control of the distance between acid groups and the behavior of ionic clusters, which makes it difficult to substantiate the mechanisms of proton transport in the phosphonated polymers obtained in this way. In [279] a controlled radical polymerization of diethyl (4-vinylphenyl)phosphonate and tetraethyl (5-vinyl-1,3-phenylene)bisphosphonate monomers with one and two phosphonate groups, respectively, was carried out (Figure 16). The resulting polymers demonstrated a significant reduction in the energy barrier for ionic conductivity due to the localization of acid groups in nanoscale domains and the formation of uniformly dispersed ionic clusters. After the addition of imidazolium ionic liquids, bisphosphonate-based polymers exhibited advantages over monophosphonate-based ones: improved conductivity, higher mechanical strength, and suppressed ionic aggregation.

**Figure 17:**
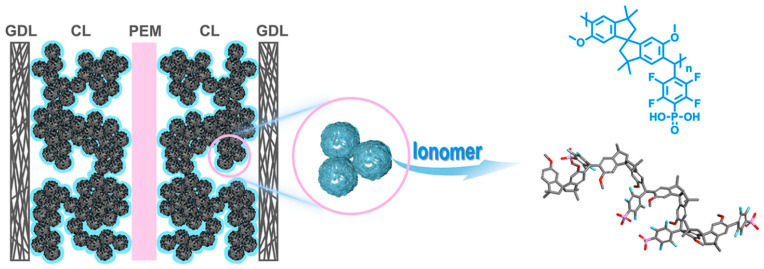
Design ideas of ionomers in PEMFCs. Reprinted with permission from [280], Copyright 2023, American Chemical Society.

In [280], a new phosphonated ionomer (PIM-P) with an intrinsic microporous structure was studied, the formation of micropores in which is due to the existence of intermolecular H-bonds and dipole forces. It was found that ionic groups involving aggregates of phosphonic acid groups exhibit a tendency to align themselves into ordered supramolecular structures (Figure 17). This results in the formation of a proton-conducting transport channel in the ionomer with high mass transfer characteristics. An analysis of gas permeability, proton conductivity, and interfacial compatibility was conducted, which, according to the authors’ conclusions, is of great importance for improving such characteristics of ionomers as mass transfer at electrodes and emphasizes the crucial role of an ordered structure for anhydrous proton conductivity in PIM. Under humidified conditions, the proton conductivity of PIM-P was 74 mS cm^−1^ at 160 °C. It was shown that the rigidity and curvature of the polymer matrix, caused by its high molecular weight, hinder proton transport.

**Figure 18:**
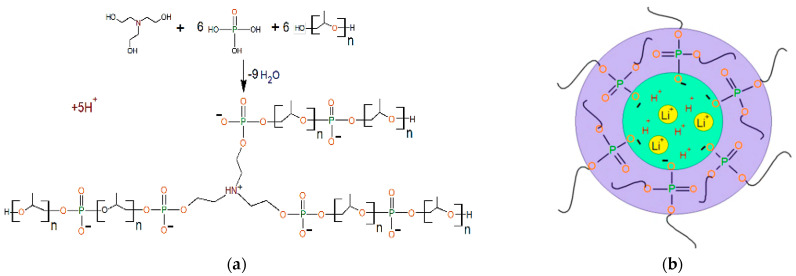
AEPA formation. (**a**) Scheme of ion-conducting channels in polymer gel electrolytes based on AEPA–PU (**b**). Reprinted with permission from [286]. Copyright 2021, Royal Society of Chemistry.

In works [281,282,283,284,285], polyurethane ionomers were obtained using aminoethers of orthophosphoric acid (AEPA) (Figure 18a) and polyisocyanates of both aromatic and aliphatic nature as the polyol component.

The influence of the content of ionogenic groups and polyphosphate structures on the electrophysical properties of the corresponding polyurethanes was analyzed. It was shown that the ionic conductivity of the polyurethane gel electrolyte synthesized on the basis of AEPA and an aromatic polyisocyanate was low (no more than 1.0 × 10^−5^ S cm^−1^ at 20 °C). The conductivity of gel electrolytes based on AEPA and aliphatic polyisocyanates increases at least 2-fold, reaching values of 6.2 × 10^−4^ S cm^−1^ at 20 °C. A feature of the supramolecular organization of AEPA-PU is the clustering of ionogenic groups, leading to the formation of cation-conducting channels (Figure 18b). The introduction of carboxylate anions into the composition of AEPA by additionally using phthalic and succinic anhydrides leads to an increase in the size of cation-conducting channels and an increase in the mobility of positively charged ions in the volume of modified AEPA-PU [286]. As a result, the conductivity of polyurethane lithium-ion gel electrolytes obtained on the basis increased to 3.0 × 10^−3^ S cm^−1^ at 20 °C. The polyurethane gel electrolyte obtained using succinic anhydride demonstrated its performance over 150 charge–discharge cycles. Moreover, the discharge capacity during the first 30 cycles changed from 450 to 140 mAh/g, then gradually decreased to a value of 82 mAh/g. The average cell potential, starting from cycle number 30, remained at 1.4 V [287].

An approach based on the esterification reaction of orthophosphoric acid also made it possible to synthesize amino ethers of boric acid (AEBA) by reacting triethanolamine, boric acid, and polyethylene oxide with an MM of 400 [288,289,290]. AEBA was shown to exist in the form of clusters, and the additional introduction of sterically hindered diols into their structure leads to a significant reduction in the particle size of AEBA and their viscosity compared to unmodified AEBA due to the partial destruction of associative interactions. Polyurethanes have been synthesized based on AEBA. It has been shown that a decrease in cluster size due to the inclusion of adducts in the AEBA structure alters the physicomechanical behavior of AEBA-PU and increases the vapor permeability of polyurethanes obtained from them by a factor of three. This effect is explained by the fact that a decrease in cluster size leads to a loosening of their dense packing. Clustering regions, due to the associative interactions of hydroxyl groups, together with the hydrophilic nature of polyethylene glycol, create channels through which water molecules can penetrate. During pervaporation separation of aqueous solutions of ethanol and isopropanol under various temperature conditions and concentration ranges, it was revealed that the permeability of composite membranes and the pervaporation index of mixture separation increase when polyurethanes obtained using modified AEBA are used as the selective layer. Membranes with a selective layer based on AEBA-PU and their modifications are hydrolytically stable and retain a set of their properties during long-term pervaporation separation [291].

## 3. Cationic Ionomers

Most commercially used ionomers contain carboxylate and sulfonate ionogenic groups [292,293]. Due to their antimicrobial activity [294,295,296], resistance to marine mollusc fouling [297], and high adhesive properties, ionomers carrying cationic functionality are attracting increasing attention. The most studied of these are cationomers containing quaternary ammonium, phosphonium, and imidazolium groups [298].

### 3.1. Imidazolium-Based Ionomers

**Figure 19:**
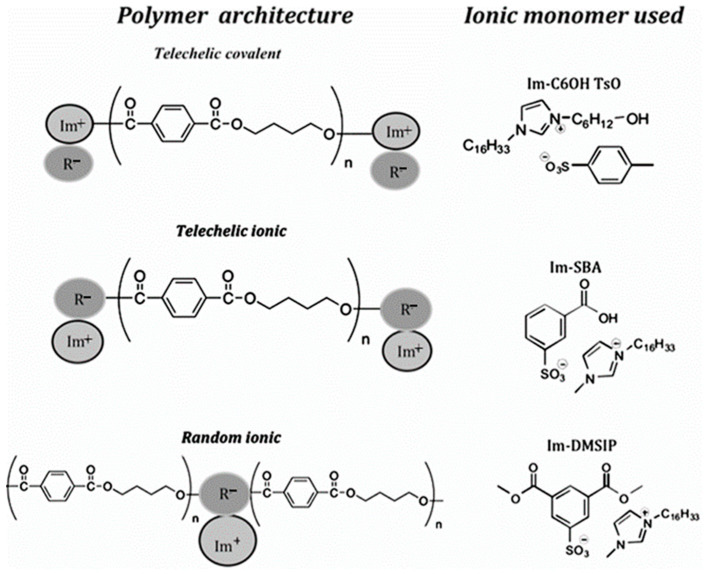
Ionomers architectures and monomers used for the synthesis where Imþ is the imidazolium salt and R is the organic anion and n is the degree of polymerization. Reprinted with permission from [308], Copyright 2012, Elsevier B.V.

Polyethylene terephthalate (PET) is a well-known fiber-forming polymer and, therefore, is widely used in the textile industry [299]. Applications of polybutylene terephthalate (PBT) are focused on electrical and electronic devices that may be used in hospitals [300]. In all these cases, the need for antimicrobial (AM) treatment to continuously suppress microbial growth is important. The most popular AM agents, such as triclosan and silver nanoparticles [301], migrate to the surface of the material during use, resulting in a decrease in AM activity over time. This circumstance has led to the incorporation of antimicrobial agents into the polymer backbone [302,303]. One method for imparting antimicrobial activity to terephthalate polyesters is based on the use of a quaternary ammonium cation to interact with the polymer backbone [304]. However, it turned out that quaternary ammonium cations decompose during the synthesis and processing of terephthalate polyesters [305]. In contrast, imidazolium salts [296,306], which do not decompose under the conditions of processing and synthesis of terephthalate polyesters, exhibit high thermal stability up to 350 °C. Imidazolium salts also exhibit high efficiency as antimicrobial agents [307]. In [308], imidazolium poly(butylene terephthalate) ionomers with ionic groups located randomly along the polymer chain or selectively as end-groups (telechelic) were obtained. To improve the long-term activity of AM, a covalent bonding and an ionic aggregation were performed to bind imidazolium to the polymer backbone and sulfonated groups covalently bonded to the polymer (Figure 19). It was shown that imidazolium ionomers retain their high antimicrobial activity after 6 days of exposure to hot water.

According to the earliest studies of ionomers [309,310], even a small amount of ionic functionality within ionomers can increase the adhesion of polymer composites. Adhesion, as well as oxidative stability and gas impermeability, are important technical properties for isobutylene-rich elastomers such as poly(isobutylene-*co*-isoprene) (BIIR) [311].

These materials are used to manufacture electrical insulating devices, vibration dampening equipment [312,313], industrial sealants, tire inner liners [314], and pharmaceutical closures. Imidazole-based nucleophiles are suitable for producing ionomer elastomers. Alkylation of imidazole nucleophiles ultimately leads to the formation of a covalent structure that is resistant to creep. The aggregation of imidazolium bromide ionic groups into clusters is the cause of the formation of a labile structure. As a result, the characteristics of the BIIR are a consequence of an unusual combination of a covalent network and an anionic network. A complete understanding of these materials requires a detailed study of both the density of the chemical polymer network and the degree of aggregation of ion pairs. It is also necessary to consider the possibility and consequences of interactions between covalent and ionic networks [315].

**Figure 20:**
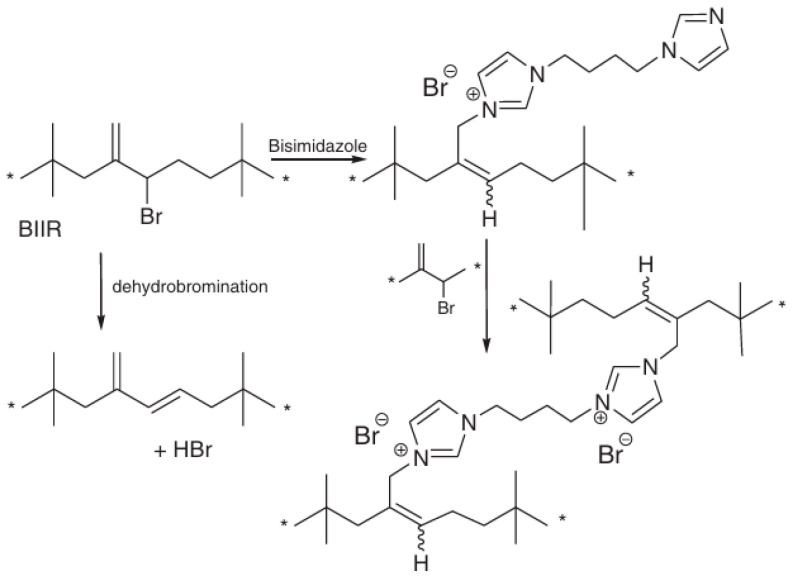
Alkylation and dehydrohalogenation reactions of BIIR to yield IIR-BisImBr. Reprinted with permission from [316], Copyright 2016, Elsevier B.V.

In [316], elastomeric ionomers were obtained by using a halide substitution reaction from brominated BIIR using imidazole-based nucleophiles (Figure 20). It was shown that the physical properties of the resulting ionomeric thermosetting elastomers are determined by the contributions of both covalent and ionic networks. Moreover, the main contribution to the dynamic storage modulus and the static elastic modulus, determined under low-strain tensile conditions, comes from the clustering of ion pairs. However, this labile network exhibits intense relaxation, which minimizes its impact over periods of more than one minute. The imidazole groups enhance the adhesive properties and antibacterial activity of these ionomers.

Poly(lactic acid) (PLA) is one of the most important and promising bioplastics derived from renewable resources [317]. However, the low strength and flexibility of PLA significantly limit its potential applications. Reference [318] demonstrated that imidazolium-based elastomeric ionomers (*i*-BIIRs) with alkyl chains of varying lengths can effectively improve the tensile elasticity of PLA. However, *i*-BIIRs were unable to increase the impact strength of PLA, which is a major challenge for poly(lactic acid) applications.

**Figure 21:**
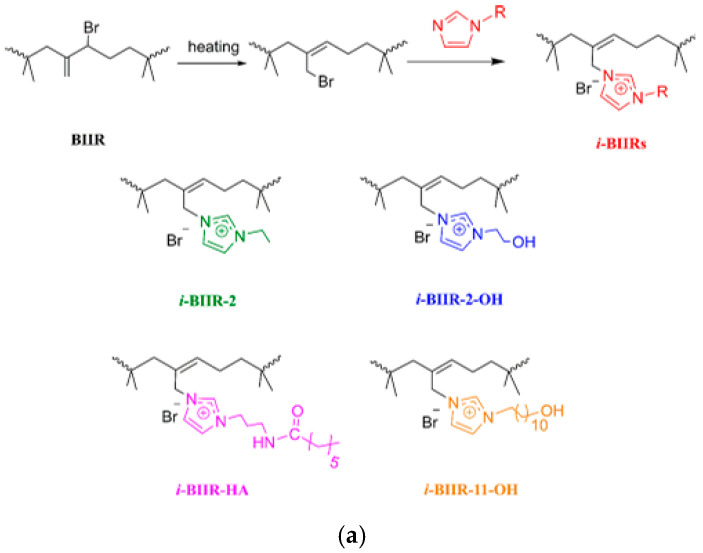

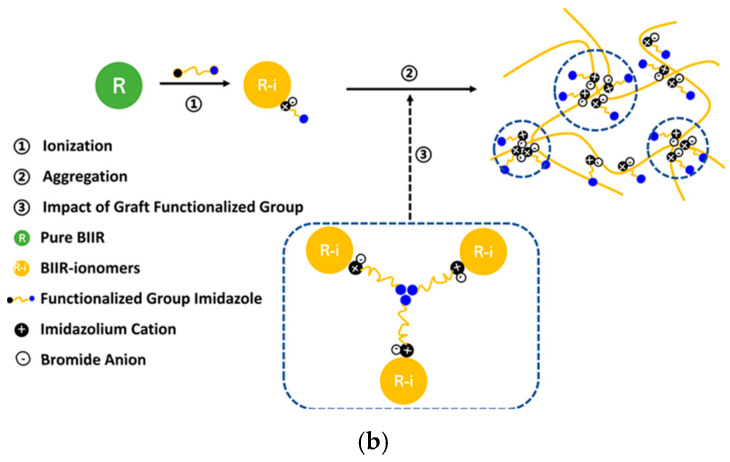
Schematic Route for Synthesizing the Imidazolium Bromide Elastomeric Ionomers (i-BIIR-2, iBIIR-2-OH, i-BIIR-HA, and i-BIIR-11-OH) and Their Structures (**a**) Hypothetical Ionic Cluster Formation Process in Various Functionalized Imidazolium-Based Grafted BIIRs. Reprinted with permission from (**b**) [319], Copyright 2020, American Chemical Society.

In [319], *i*-BIIRs were investigated as modifiers for PLA. It turned out that in the case of using alkyls terminated with an amide or hydroxyl group in the imidazolium cation of *i*-BIIR (Figure 21), an increase in compatibility, impact strength, elongation at break and an optimal ratio of flexibility and rigidity for PLA/*i*-BIIR ionomer blends is observed due to an improvement in the interfacial adhesion between the evenly dispersed small ionomer and poly(lactic acid).

As shown in the introduction, the development of shape-memory materials is largely associated with dynamic and supramolecular polymers, in which the action of an external force can lead to reversible rupture and subsequent restoration of existing interactions within the polymer [320,321]. Such interactions include chemical or physical cross-links that undergo reversible changes under certain temperature conditions [255,322,323].

Under these conditions, the spatial polymer network of covalent cross-links performs the function of maintaining the memory of the initial state, and the ionic network, existing as a result of clustering, undergoes reversible rupture and restoration after deformation. The main factor determining shape restoration is the relaxation of polymer chains of an entropic nature [324,325]. Shape memory materials are finding application in the fabrication of deployable and morphing structures, medical implants, actuators, and self-healing systems [326,327,328,329,330].

One approach to developing shape memory materials is based on organic-inorganic hybrids in which a soft polymeric canopy is bound to a well-defined nanoparticle core by ionic interactions [331,332,333,334]. Such polymers have been proposed as nanoscale ionic materials (NIMs) because their design incorporates nanometer-sized inorganic cores whose surfaces are coated with anions, while low-molecular-weight polymers serve as counterions. Poly(ethylene oxides) with terminal amino groups can be used for this purpose. The hybrid nature of the NIMs creates the possibility of rapid exchange between ionically modified nanoparticles and the polymeric canopy and targeted control of their properties by selectively selecting components [335]. The use of surface-functionalized nanoparticles is attracting increasing attention from researchers and is leading to the development of new hybrid polymer materials [336].

**Figure 22:**
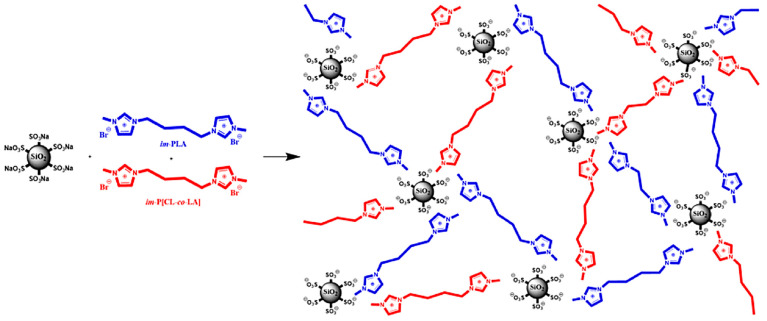
Schematic of Ionic Hybrids Based on PLA and Imidazolium-Terminated PLA and P[CL-co-LA] Oligomers with Sulfonated Silica Nanoparticles. Reprinted with permission from [337], Copyright 2017, American Chemical Society.

In [337], the reversibility of ionic bonds in NIMs, combined with the reinforcement effect of the nanoparticles, was used. The preparation of NIMs in this work is based on hybrids of blends of imidazolium-terminated oligomers of glassy polylactide (PLA) and rubbery poly[ε-caprolactone-*co*-D,L-lactide] (P[CL-*co*-LA]) (im-P[CL-*co*-LA]) dispersed in commercial PLA with sulfonate-modified silica (Figure 22). The dynamic nature of reversible ionic interactions makes it possible to transform a polymer (in this case, commercial PLA) into a polymer exhibiting a unique property called shape memory.

**Figure 23:**
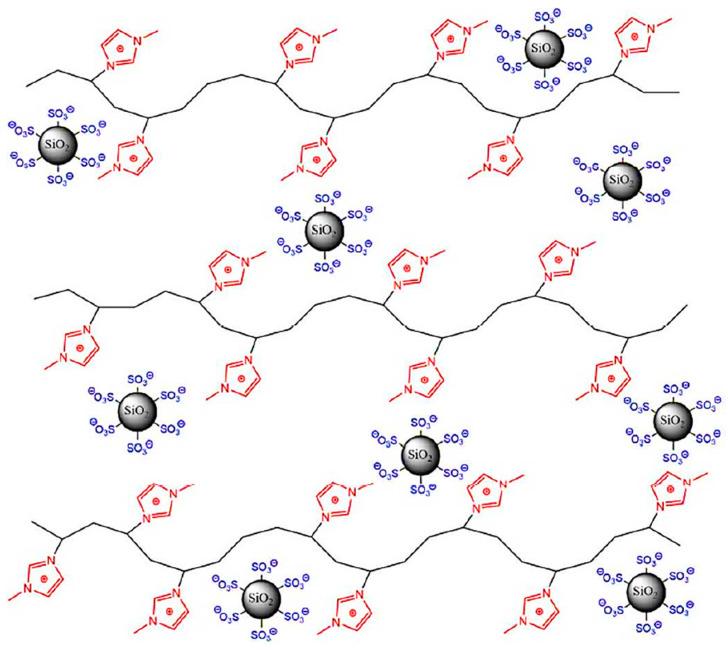
Ionic nanocomposites synthesized via self-assembly of imidazolium-functionalized polyurethane with sulfonate-modified silica nanoparticles. Reprinted with permission from [338], Copyright 2017, Royal Society of Chemistry.

Sulfonated silicon nanoparticles in combination with polyurethanes containing imidazolium groups in the side branches (im-PU) were used to synthesize polyurethane NIMs (Figure 23) [338]. It was shown that with an increase in the content of sulfonated silicon nanoparticles, the polyurethanes change rheologically, undergoing a transition from liquid-like to solid-like behavior. The presence of ionic interactions, which impart additional branches of an ionic nature, makes it possible to disperse silica nanoparticles at high contents in polyurethanes. As a result, it turned out that with a content of 20 wt.% sulfonated silicon nanoparticles, it was possible to achieve a forty-fold increase in tensile strength and a more than ten-fold increase in elongation at failure of the sample. At the same time, the rigidity of the samples increases by 2.5 times compared to unmodified polyurethane. Polyurethane NIMs, due to the existence of ionic interactions in their polymer matrix, are completely restored even after exposure of samples to significant deformation forces, demonstrating the property of self-healing.

**Figure 24:**
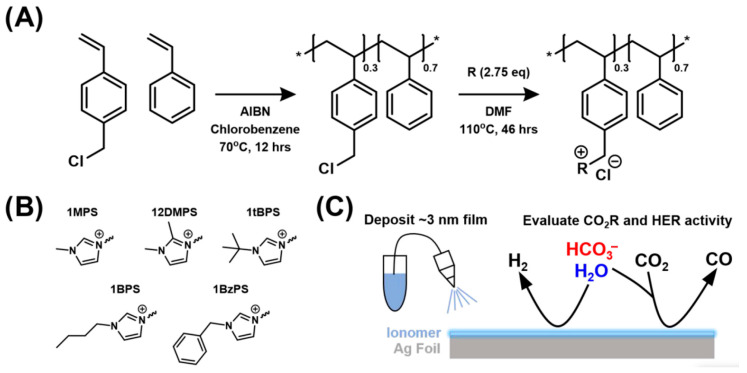
(**A**) Synthetic scheme for imidazolium ionomers. (**B**) Structures for the five imidazolium groups tested, connected to the polystyrene backbone at the wavy bond and with abbreviations denoted above corresponding structure. (**C**) Schematic of experimental approach showing deposition of a thin ionomer film on Ag foil by spray coating and subsequent CO_2_R testing. Reprinted with permission from [351], Copyright 2021, American Chemical Society.

Ionomers have proven useful in the field of heterogeneous catalysis due to their ability to link electrocatalyst layers and facilitate ion transport [339], and much of the development has focused on optimizing either cationic or anionic conductivity [340,341]. The direct involvement of ionomers in catalytic processes has been best studied in oxygen reduction reaction (ORR) processes, where perfluorosulfonic acid polymers (e.g., Nafion) electrostatically adsorb to Pt surfaces and reduce the available surface area for catalysis [342]. In catalytic processes, ionomers necessarily form interfaces with catalyst surfactants. As a result of this interaction, ionomers become involved in the reaction layer, and noncovalent interactions with reactants could tune kinetics [343,344,345,346]. In [347,348], the catalytic properties of ionomers in which imidazolium cations are attached to a polystyrene base were studied, promoting the reduction of CO_2_R to CO through specific noncovalent interactions with CO_2_ or CO_2_R intermediates [349,350]. High selectivity for the electrochemical reduction of CO_2_R to CO on Ag surfaces was demonstrated when used as a membrane in membrane electrode assembly tests. It was noted that studying the interaction between the surfaces of catalytic systems and ionomers makes it possible to increase the reaction activity and control its selectivity. In [351], imidazolium-based ionomers were studied in the electrocatalytic reduction of CO_2_ to CO (CO_2_R) (Figure 24). The reaction was carried out on a silver surface. It was found that imidazolium ionomers accelerate the competing hydrogen evolution reaction (HER), and a mechanism for their catalytic action was proposed using Taft steric parameters and density functional theory calculations.

### 3.2. Quaternary Ammonium-Based Ionomers

One of the advantages of using bulk quaternary ammonium counterions is the improvement of the hydrophobicity of sulfonated monomers [352]. This makes it possible to copolymerize them with nonpolar comonomers in organic media and to use controlled radical polymerization [353,354,355]. The use of bulk quaternary ammonium counterions makes it possible to modify the thermal and viscoelastic properties of sulfonated polystyrene [356,357,358] and polyesters [359], phosphonium-containing polysiloxanes [360], and poly(oligo(ethylene glycol) methyl ether methacrylate-co-styrenesulfonate) [361]. Ionic clusters in these polymers create a physical network that effectively enhances polymer–polymer interactions. At the same time, the characteristic relaxation times of polymers increase, and electrostatic interactions determine the dissociation-association lifetime, the mobility of the ion pair, and the possibility of ion exchange between clusters. This phenomenon, also known as ion hopping, can affect the linear viscoelastic response of the polymer [251,362,363].

**Figure 25:**
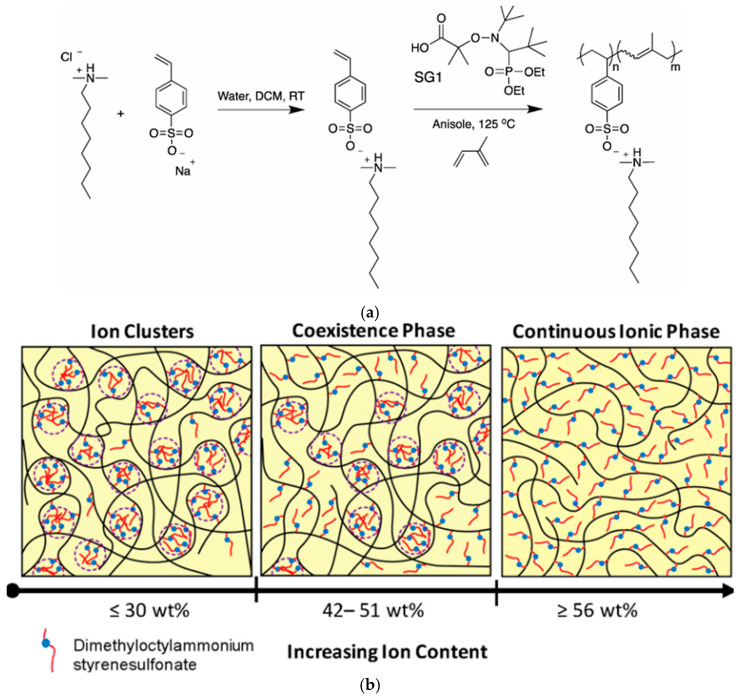
Salt Metathesis Reaction and Subsequent Nitroxide-Mediated Polymerization of DMOASS with Isoprene (**a**) and three modes of polymer matrix formation depending on the DMOASS content (**b**). Reprinted with permission from [366], Copyright 2018, American Chemical Society.

The use of bulkier counterions, such as alkylammonium or phosphonium, leads to the possibility of slight screening of electrostatic interactions and, as a consequence, dissociation of ion clusters at lower temperatures, a decrease in the glass transition temperature and melt viscosity [364,365]. In the work [366], poly(isoprene-*ran*-dimethyloctylammonium styrenesulfonate) (P(I-*ran*-DMOASS)) copolymers of varying dimethyloctylammonium styrenesulfonate (DMOASS) compositions (30−77 wt.%) were synthesized. Poly(isoprene-*ran*-dimethyloctylammonium styrenesulfonate) (P(I-*ran*-DMOASS)) ionomers with controlled ion content were synthesized by nitroxide-mediated direct copolymerization of dimethyloctylammonium styrenesulfonate (DMOASS) with isoprene. Three structural regimes were identified in the series of ionomers obtained (Figure 25). In the ion-cluster structure (30 wt.% DMOASS), the glass transition temperature of P(I-*ran*-DMOASS was significantly affected by the isoprene matrix and, due to the existence of clusters, increased connectivity between the polymer chains was observed. At intermediate DMOASS contents (42−51 wt.%), the dynamic mechanical analysis curves showed the appearance of two Tg regions in DMA, resulting from the coexistence of both structures. At upon transition to the continuous ionic phase regime (56−77 wt.% DMOASS), the copolymers behaved as entangled macromolecules as a result of electrostatic interactions. As a result, melt flowability was limited, and polymer elasticity decreased.

**Figure 26:**
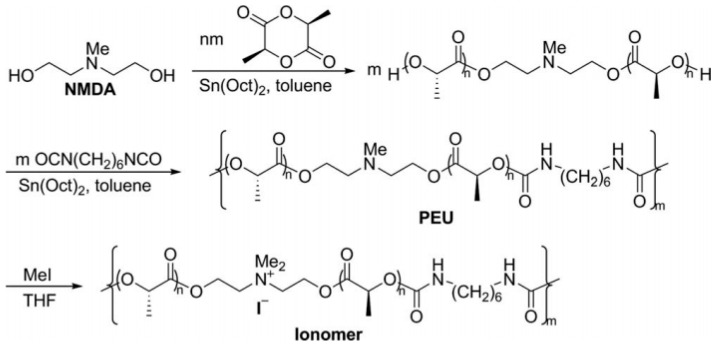
Preparation of PLLA-based cationic ionomers. Reprinted with permission from [367], Copyright 2013, Wiley.

In [367], a polyurethane cationomer was synthesized based on polylactide and N-methyldiethanolamine with the addition of methyl iodide, 2-ethylhexanoate of tin (II) and hexamethylene diisocyanate (Figure 26). The introduction of tertiary amino groups into the composition of polyurethane based on polylactide and their ionization had an effect on the physical and mechanical properties of PU, on hydrophilicity and the ability to biodegrade while maintaining the relatively high crystallinity of polylactide.

### 3.3. Prospects for the Synthesis Strategy of Cationic Ionomers

Achieving high levels of microphase separation and high strength properties of ionomers is possible by combining ionic interactions with the block copolymer architecture of the polymers. Polyurethane ionomers are promising in this area [368,369]. In cases where ionic groups cause phase separation and maintain a physically cross-linked network, constructing a structure similar to random ionomers for block copolymers offers potential.

**Figure 27:**
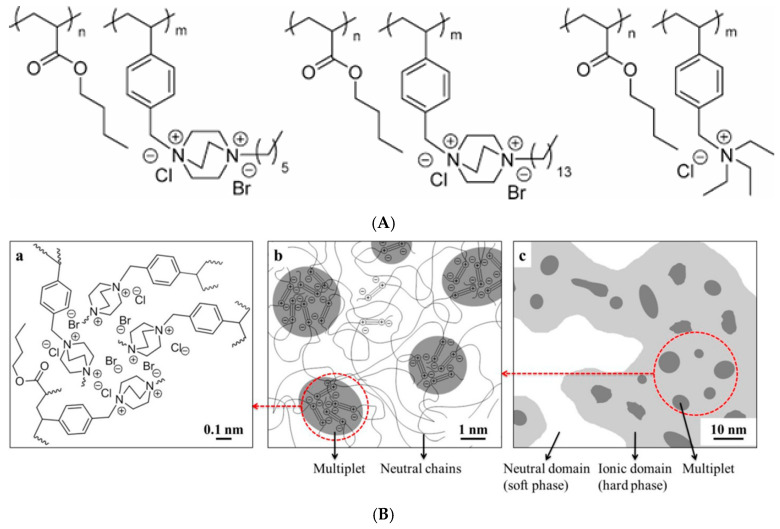
Chemical structures of poly(VBDC_6_BrCl-co-nBA), poly(VBDC_14_BrCl-co-nBA), and poly(VBTEACl-co-nBA) random copolymers (**A**) pictorial representation for aggregation of DABCO salt units, multiplets, and ionic domains of poly(VBDCxBrCl-co-nBA) with halide counterions (**B**). (**a**) aggregation of ions driven by dipole–dipole (columbic) interactions, (**b**) spatial arrangement of multiplets, note some ion pairs not able to associate with a specific multiplet, (**c**) spatial heterogeneity of ionic domains (hard phase) containing a high density of ions and multiplets dispersed with neutral domains (soft phase). Reprinted with permission from [378]. Copyright 2016, American Chemical Society.

To optimize the properties of these polymers, it is necessary to establish the relationships between the structure and properties of random ionomers. In this regard, a design feature of ion-containing polymers is based on the use of monomers carrying more than one ionic center [370,371,372,373]. According to these studies, multiply charged monomers allow for more effective improvement of polymer properties compared to singly charged control samples. Such macromolecular systems include, in particular, polymers containing zwitterions [374,375,376]. In [377], random copolymers with a pair of ammonium cations on each ionic pendant group were obtained by copolymerization of *n*-butyl acrylate (*n*BA) and styrene monomers containing quaternized diazabicyclo[2.2.2]octane (DABCO) groups. Comparison of the properties of these polymers with control samples of random copolymers containing a single-charged trialkylammonium group demonstrated similarity in thermal stability and thermal transitions, but significant differences in thermomechanical properties. In the work ([378] random copolymers containing diazabicyclo[2.2.2]octane salt-containing random copolymers with two quaternized nitrogen cations on each ionic pendant group were synthesized. Copolymers with a random distribution of monomer units containing triethyl-(4-vinylbenzyl)ammonium chloride were used as singly charged controls (Figure 27). It was found that divalent DABCO salts contributed to a more distinct microphase-separated morphology in comparison with singly charged analogs and higher thermomechanical and strength properties of the ionomers.

Molecules that contain covalently bonded cations and anions in equal amounts are defined as zwitterions (also called polybetaines) [370], and polymers containing functional zwitterions as pendant groups are referred to as polyzwitterions. To achieve structural diversity of both the backbone and the zwitterions, polyzwitterions are prepared by various methods, including chain and step-polymerization of zwitterionic monomers and post-polymerization modification. There are also strategies for generating zwitterionic functionality directly on macromolecular structures [379]. This is accomplished by alkylation of tertiary amine-containing repeat units of polymer chains with anionic or ionizable ligands. A wide range of zwitterionic structures are known, including various combinations of anions (sulfonates, carboxylates, sulfates, phosphonates, phosphates, phosphinates) and cations (imidazoliums, phosphoniums, ammoniums) [380,381,382,383,384,385].

**Figure 28:**
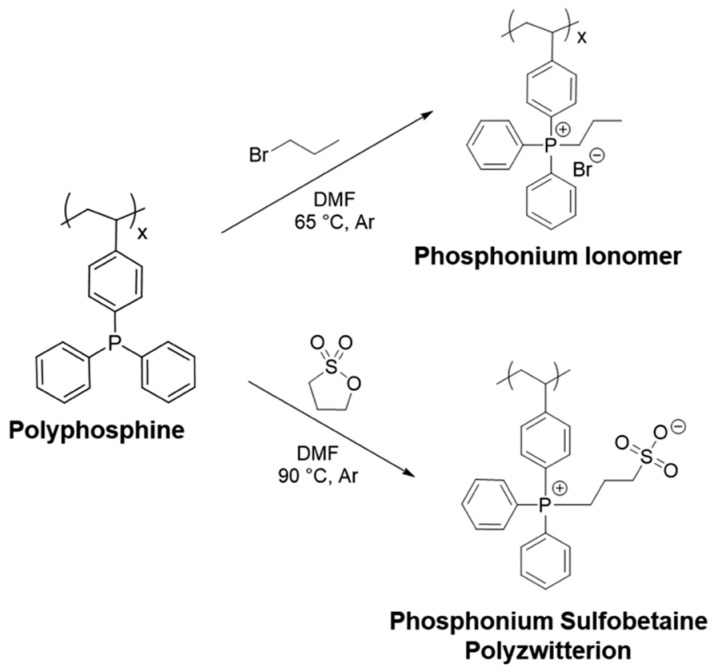
Alkylation Strategies for the Synthesis of Phosphonium-Based Polyelectrolytes and Polyzwitterions from polyDPPS Homopolymer Precursors. Reprinted with permission from [393]. Copyright 2020, American Chemical Society.

Polyzwitterionic materials can exhibit functions such as biomimicry [386,387], the ability to self-assemble [375,388], conjugate drugs [389], thermomechanical reinforcement [376,390], and prevent microbial and biofouling [391]. Zwitterions, compared to conventional ion pairs, have larger dipole moments and, due to this, provide stronger physical crosslinking compared to other ionomers [392]. In [393], unique phosphonium-based zwitterionic homopolymers and random copolymers were obtained and studied. The synthesis strategy included free-radical polymerization of 4-(diphenylphosphino)styrene followed by the preparation of neutral polymers containing reactive triarylphosphines. Postpolymerization alkylation of the pendant functionalities was then carried out. As a result, polymers containing various concentrations of neutral phosphines, phosphonium ions, and phosphonium sulfobetaine zwitterions were obtained (Figure 28). Due to nanoscale morphological domains, which formed due to electrostatic interactions between zwitterionic groups, the resulting polymers differed from neutral and phosphonium analogs by the significantly higher glass transition temperatures and enhanced mechanical reinforcement.

One promising method for creating covalent adaptable networks (CANs) is the use of transalkylation reactions. In [394,395,396], polyionic polymer networks were obtained using triazolium. The resulting alkyl triazolium then participates in dissociative exchange, leading to the repeated formation of cross-links, the apparent cross-link density of which remained almost constant over a wide temperature range. Covalent adaptable networks were also obtained using the transalkylation exchange reaction of quaternized amino-based salts, such as anilinium and pyridinium salts [397,398,399]. In [400], the dynamic network was designed using a divalent cross-linker, 1,4-diazabicyclo[2.2.2]octane. This compound was obtained using quaternary ammonium salts as a transalkylation-based dynamic covalent chemistry platform. A similar approach using the transalkylation of C-N bonds in PU was implemented by researchers in [401,402]. This resulted in ion-conducting polyurethanes. The transalkylation reaction was used in the development of sustainable solid-state electrolytes [403]. Even after undergoing multiple healing processes, the resulting vitrimer ionogel materials maintained their conductive behavior.

**Figure 29:**
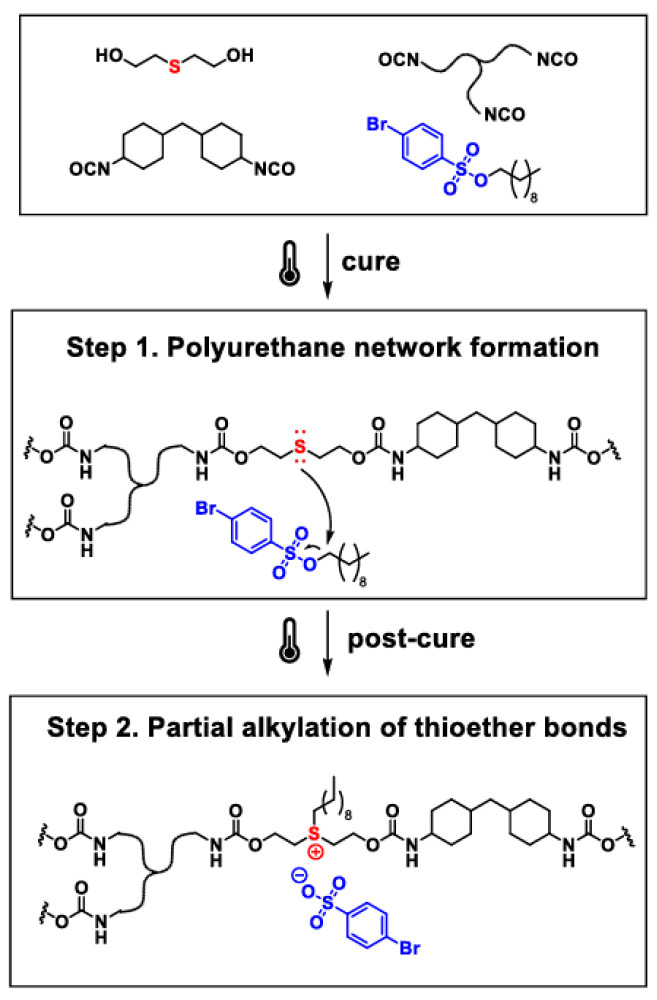
Schematic Overview of the Synthetic Protocol toward PU Networks Containing Trialkylsulfonium Salts. Reprinted with permission from [404]. Copyright 2023, American Chemical Society.

In [404], polyurethane coatings were obtained by partial alkylation of the thi-oether bonds (Figure 29), yielding dynamic trialkylsulfonium bonds that are able to exchange via transalkylation at elevated temperatures. In the cured form, the glass transition temperature of these coatings reaches 70 °C; they exhibit high transparency, minimal coloration, and property retention after multiple processing cycles.

## 4. Discussion and Outlook

An important feature of the pendant ionic groups of ionomers is their tendency to sequentially combine into multiplets and then into cluster supramolecular ionic formations. This results in thermally reversible microphase separation of the clusters and the non-polar polymer phase. The reversibility of microphase separation and the underlying processes of intermolecular interactions and reversible cross-linking determine the fundamental properties and applications of ionomers. The ionic functions of ionomers are determined primarily by the type of ions (cations or anions) that serve as the basis for their functionalization. Thus, ionomers containing anionic pendant ions are anionic ionomers, while ionomers containing cationic pendant ions should be classified as cationic ionomers.

Sulfonate, carboxylate, acrylate, and phosphonium groups are used to develop anionic ionomers. Neutralization of anionic pendant ions can be accomplished using monovalent counterions such as alkali metal ions and organic alkyl ammonium ions. Dipole–dipole interactions involving ion pairs lead to the formation of a physical polymer network. However, multivalent counterions, which are capable of forming ionic bonds between two monovalent pendant cations, offer a wider range of Coulombic interactions and the potential for stronger physical cross-linking. In the case of anionomers, alkaline earth ions and transition metal ions can be used as multivalent counterions.

Some of the most representative cationic ionomers are polymers with imidazolium, quaternary ammonium, and pyridinium functional groups. Multivalent pendant cations are also known (e.g., 1,4-diazabicyclo[2.2.2]octane (DABCO) double ammonium salt). Polyzwitterionic materials, in particular phosphonium-based zwitterionic homopolymers and random copolymers, are promising. To neutralize monovalent side cations, monovalent pendant ions such as halides and tosylate anions are usually used. Currently, in the development of cationomers, more and more work is aimed at the use of multivalent counterions.

## Data Availability

No new data were created or analyzed in this study.
